# Early Hyperbaric Oxygen Treatment Attenuates Burn-Induced Neuroinflammation by Inhibiting the Galectin-3-Dependent Toll-Like Receptor-4 Pathway in a Rat Model

**DOI:** 10.3390/ijms19082195

**Published:** 2018-07-27

**Authors:** Zong-Sheng Wu, Jing-Jou Lo, Sheng-Hua Wu, Chau-Zen Wang, Rong-Fu Chen, Su-Shin Lee, Chee-Yin Chai, Shu-Hung Huang

**Affiliations:** 1 Department of Medical Laboratory Science and Biotechnology, College of Health Sciences, Kaohsiung Medical University, 807 Kaohsiung, Taiwan; a8905114@gmail.com; 2 School of Post-Baccalaureate Medicine, College of Medicine, Kaohsiung Medical University, 807 Kaohsiung, Taiwan; joekll@hotmail.com; 3 Department of Anesthesiology, Kaohsiung Medical University Hospital, 807 Kaohsiung, Taiwan; elsawu2@gmail.com; 4 Department of Anesthesiology, Kaohsiung Municipal Hsiao-Kang Hospital, Kaohsiung Medical University, 807 Kaohsiung, Taiwan; 5 Graduate Institute of Medicine, College of Medicine, Kaohsiung Medical University, 807 Kaohsiung, Taiwan; czwangkmu00@gmail.com; 6 Department of Physiology, College of Medicine, Kaohsiung Medical University, 807 Kaohsiung, Taiwan; 7 Department of Medical Research, Kaohsiung Medical University Hospital, 807 Kaohsiung, Taiwan; 8 Orthopaedic Research Center, College of Medicine, Kaohsiung Medical University, 807 Kaohsiung, Taiwan; 9 Division of Plastic Surgery, Department of Surgery, Kaohsiung Medical University Hospital, Kaohsiung, Taiwan, 807 Kaohsiung, Taiwan; dr.chenrf@gmail.com (R.-F.C.); sushin@kmu.edu.tw (S.-S.L.); 10 Department of Surgery, School of Medicine, College of Medicine, Kaohsiung Medical University, 807 Kaohsiung, Taiwan; 11 Department of Pathology, Kaohsiung Medical University Hospital, Kaohsiung Medical University, 807 Kaohsiung, Taiwan; ccjtsai@yahoo.com; 12 Hyperbaric Oxygen Therapy Room, Kaohsiung Medical University Hospital, Kaohsiung Medical University, 807 Kaohsiung, Taiwan

**Keywords:** hyperbaric oxygen, neuroinflammation, burn, galectin-3, toll-like receptor-4

## Abstract

Hyperbaric oxygen (HBO) treatment has been proven to decrease neuroinflammation in rats. This study aimed to determine the potential mechanism underlying the anti-inflammatory effects of HBO treatment on burn-induced neuroinflammation in rats. Thirty-six adult male Sprague-Dawley (SD) rats were randomly assigned to the following six groups (*n* = 6 per group): (1) sham burn with sham HBO treatment; (2) sham burn with HBO treatment; (3) burn with one-week sham HBO treatment; (4) burn with two-week sham HBO treatment; (5) burn with one-week HBO treatment; and (6) burn with two-week HBO treatment. SD rats that received third-degree burn injury were used as a full-thickness burn injury model. Subsequently, we analyzed the expression of proteins involved in the galectin-3 (Gal-3)-dependent Toll-like receptor-4 (TLR-4) pathway through enzyme-linked immunosorbent assay (ELISA), immunohistochemistry (IHC) analysis, and Western blotting. A behavior test was also conducted, which revealed that HBO treatment significantly suppressed mechanical hypersensitivity in the burn with HBO treatment group compared to the burn with sham HBO treatment group (*p* < 0.05). ELISA results showed that tumor necrosis factor α (TNF-α) and interleukin 1 beta (IL-1β) levels in the dorsal horn of the spinal cord and the skin significantly decreased in the burn with HBO treatment group compared with the burn with sham HBO treatment group (*p* < 0.05). Western blotting results demonstrated that HBO treatment significantly reduced the expression of Gal-3 and TLR-4 in the dorsal horn of the spinal cord in the burn with HBO treatment group compared with the burn with sham HBO treatment group (*p* < 0.05). IHC analysis showed that the expression of Gal-3, TLR-4, CD68 and CD45 in the dorsal horn of the spinal cord was significantly lower in the burn with HBO treatment group than in the burn with sham HBO treatment group (*p* < 0.05), and the expression of CD68 and macrophage migration inhibitory factor (MIF) in the right hind paw skin was significantly lower. The expression of vimentin and fibroblast growth factor in the right hind paw skin was significantly higher after HBO treatment (*p* < 0.05). This study proved that early HBO treatment relieves neuropathic pain, inhibits the Gal-3-dependent TLR-4 pathway, and suppresses microglia and macrophage activation in a rat model.

## 1. Introduction

Neuroinflammation is connected to the activation of microglia [1]. Microglial cells are the primary macrophages of the central nervous system (CNS), and these cells modulate neuroinflammation and neuronal death [2]. Exposure to extracellular toxins, pathogens, and lipopolysaccharides (LPSs) through cell surface receptors (e.g., Toll-like receptor-4 (TLR-4) and TLR-2) activates microglial cells, increasing their production of proinflammatory cytokines, chemokines, and reactive oxygen species (ROS) [3,4,5]. A study found that suppressing microglial cell activation may decrease the severity of neuropathological disease [2].

Hyperbaric oxygen (HBO) therapy is a low-cost medical treatment with few side effects and uses reusable equipment. HBO has been commonly applied for many diseases; in this therapy, patient breathes 100% oxygen at more than 1.4 atmosphere absolute (ATA) [6]. HBO therapy is performed in a special chamber in which the entire space is pressurized with 100% oxygen, so the patient directly breathes pressured oxygen in the chamber or the pressured oxygen can be given through masks, head hoods, or endotracheal tubes. HBO treatment exerts neuroprotective effects through various mechanisms, including the inhibition of inflammation, reduction of hypoxia, and improvement of microcirculation in the nervous system [7]. Furthermore, HBO treatment reduced the levels of inflammatory cytokines, IL-1β and tumor necrosis factor-α (TNF-α), in a rat model [8,9,10,11]. HBO therapy is also applied for many types of central or peripheral nerve injury diseases. HBO treatment can promote the recovery of injured nerves and the regeneration of peripheral nerves by improving tissue oxygen supplementation. The anti-inflammatory effects of HBO therapy have been demonstrated in an animal model [12,13,14,15,16].

A study reported that the galectin-3 (Gal-3) and TLR-4 interaction is related to neuroinflammation in a mouse model [17]. Currently, 14 mammalian galectins have been reported, and they can be divided into three groups: prototype (Gal-1, -2, -5, -7, -10, -11, -13 and -14), chimera (Gal-3), and tandem repeat (Gal-4, -6, -8, -9 and -12). Galectins are involved in many physical processes and pathological responses, including cell proliferation, cell adhesion, cell apoptosis, cell activation, and phagocytosis [18,19,20,21].

Gal-3 is a pleiotropic protein, and its effects depend on its subcellular location (cell surface, cytoplasm, nucleus, endosomal compartment, and mitochondria). Thus, this protein can react to various scenarios [22,23]. Previous studies identified Gal-3 as a proinflammatory protein that regulates immune responses [24,25,26]. Gal-3 is associated with inflammatory diseases such as systemic lupus erythematosus, rheumatoid arthritis, and systemic sclerosis, and Gal-3 inhibition is associated with neuropathic pain attenuation [27,28,29,30]. Gal-3 also modulated TLR pathways in synovial fibroblasts [31].

TLRs recognize microorganisms and endogenous damage-associated signals, and they play a role in the activation of innate immune defenses. TLRs also connect innate and adaptive immunity through NF-κB, c-Jun N-terminal kinase, and p38 MAP (mitogen-activated protein) kinase pathways, and they recruit immune cells [32,33]. Experimental evidence showed that TLRs are expressed in a wide range of cell types, and the receptors contribute to the systemic inflammatory response and the induction of cytotoxicity against tumors [34]. TLR-4 also recognizes many proteins and lipoproteins, and regulates the release of proinflammatory cytokines [35,36,37,38]. Thus, the inhibition of the TLR-4 signaling pathway is associated with neuroprotective and anti-inflammatory effects [39].

In our previous study [40], we found that full-thickness burn injury causes peripheral nerve damage and induces microglial cell activation. HBO treatment was proven to inhibit inflammation [12]. We hypothesized that the Gal-3-dependent TLR-4 pathway is involved in burn injury-induced neuroinflammation. Moreover, no study has investigated the effects of HBO treatment on an animal model of burn-induced neuroinflammation. This study aimed to determine the potential mechanism underlying the anti-inflammatory effects of HBO treatment on burn-induced neuroinflammation in rats.

## 2. Results 

### 2.1. HBO Treatment Ameliorates Burn-Induced Mechanical Allodynia

**Figure 1:**
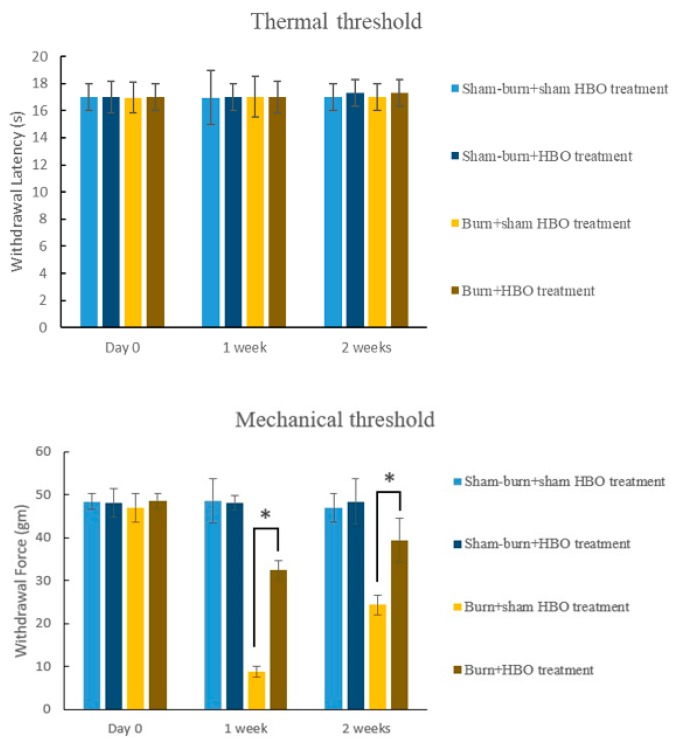
Mechanical withdrawal threshold (MWT) and thermal withdrawal latency (TWL) in each group (*n* = 6 rats per group). No change in thermal hyperalgesia was observed between groups. The MWT increased significantly in the burn with hyperbaric oxygenation (HBO) treatment group compared with the burn with sham HBO treatment group. * *p* < 0.05; third-degree burn injury.

Our rat model of burn injury was based on the modifications of similar models used in previous studies [35]. The mechanical withdrawal threshold (MWT) significantly decreased in the burn with sham HBO treatment group. The hot metal surface-caused burn injury resulted in mechanical allodynia. No significant change occurred in the thermal withdrawal latency (TWL) in the sham burn group or in the burn with sham HBO treatment group (Figure 1). The MWT (g) increased significantly in the HBO treatment groups compared with the burn with sham HBO treatment group. In rats with burn injury, the MWT (g) almost reached normal levels after HBO treatment for two weeks. Overall, the MWT (g) results demonstrated that HBO treatment improved full-thickness burn injury-induced pain.

### 2.2. HBO Treatment Inhibits Microglial Cell Activation and Reduces Proinflammatory Cytokine Expression and Macrophage Recruitment

**Figure 2:**
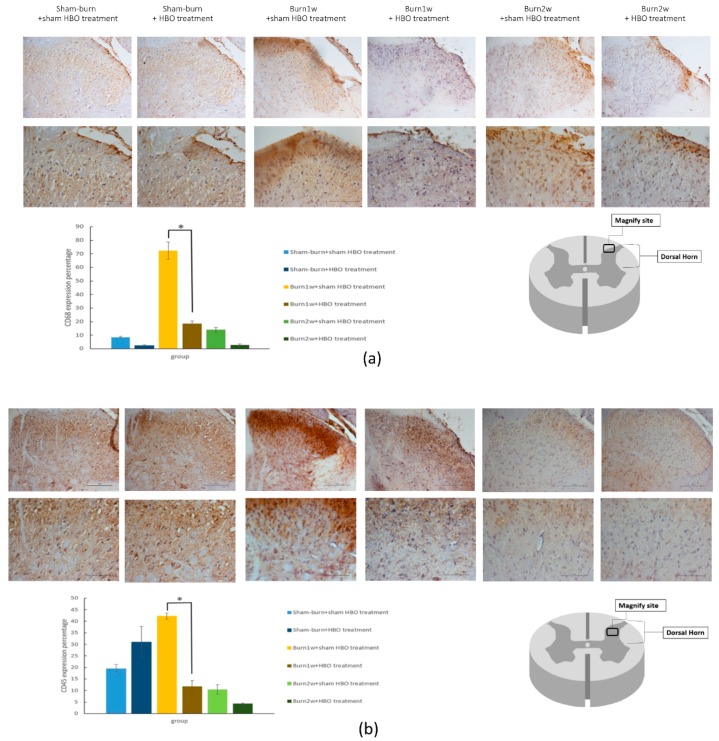
(**a**,**b**) HBO treatment inhibited CD68 and CD45 expression in spinal cord right dorsal horn histologic specimens. Immunohistochemistry (IHC) analyses of CD68 and CD45 in the spinal cord dorsal horn at 1 and 2 weeks after burn injury. CD68 and CD45 expression decreased significantly in the burn with HBO treatment group compared with the burn with sham HBO treatment group. * *p* < 0.05, original magnification: ×20 for upper part in (**a**,**b**), original magnification: ×40 for lower part in (**a**,**b**).

**Figure 3:**
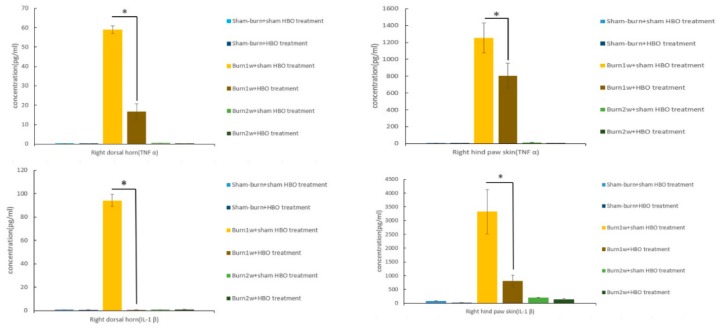
ELISA test. HBO treatment significantly inhibited the expression of the inflammatory proteins TNF-α and IL-1β in the dorsal horn of the spinal cord and the right hind paw skin in the burn with one-week HBO treatment group compared with the burn with sham HBO treatment group. * *p* < 0.05.

**Figure 4:**
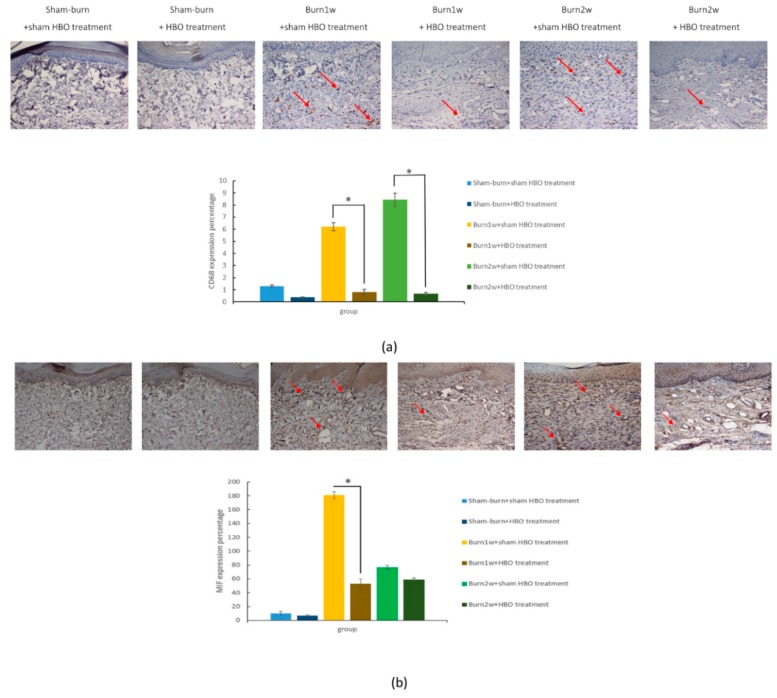
(**a**,**b**) HBO treatment decreased CD68 and macrophage migration inhibitory factor (MIF) expression. Immunohistochemistry (IHC) analyses of CD68 and macrophage migration inhibitory factor (MIF) in hind paw skin histologic specimens at one and two weeks after burn injury. CD68 expression decreased significantly in the burn with HBO treatment group compared with the burn with sham HBO treatment group. MIF expression decreased significantly in the burn with one-week HBO treatment group compared with the burn with one-week sham HBO treatment group. * *p* < 0.05, original magnification: ×20. The arrows indicate nuclei with CD68 and MIF expression.

To determine the relationship between HBO treatment and inflammatory cytokines, immunohistochemistry (IHC) analysis and ELISA were used to evaluate protein expression in the right dorsal horn and skin tissues. The expression of CD45 and CD68 in the right dorsal horn significantly decreased in rats with burn injury that received HBO treatment for one week (Figure 2a,b). Moreover, the expression of the proinflammatory cytokines TNF-α and IL-1β significantly decreased in the burn with HBO treatment group. HBO treatment significantly attenuated inflammatory protein levels in the dorsal horns of the spinal cord and the right hind paw skin (Figure 3). The IHC analysis results of CD68 and macrophage migration inhibitory factor (MIF) revealed less macrophage accumulation in the right hind paw skin in the burn with HBO treatment group (Figure 4a,b). These results suggest that HBO treatment reduces the expression of the proinflammatory cytokines TNF-α and IL-1β by inhibiting microglial cell and macrophage activation and accumulation in the right dorsal horn. 

### 2.3. HBO Treatment Inhibits Gal-3 and TLR-4 Expression in the Dorsal Horns of the Spinal Cord and Gal-3 and TLR-4 Immunohistochemical Localization in the Hind Paw Skin

**Figure 5:**
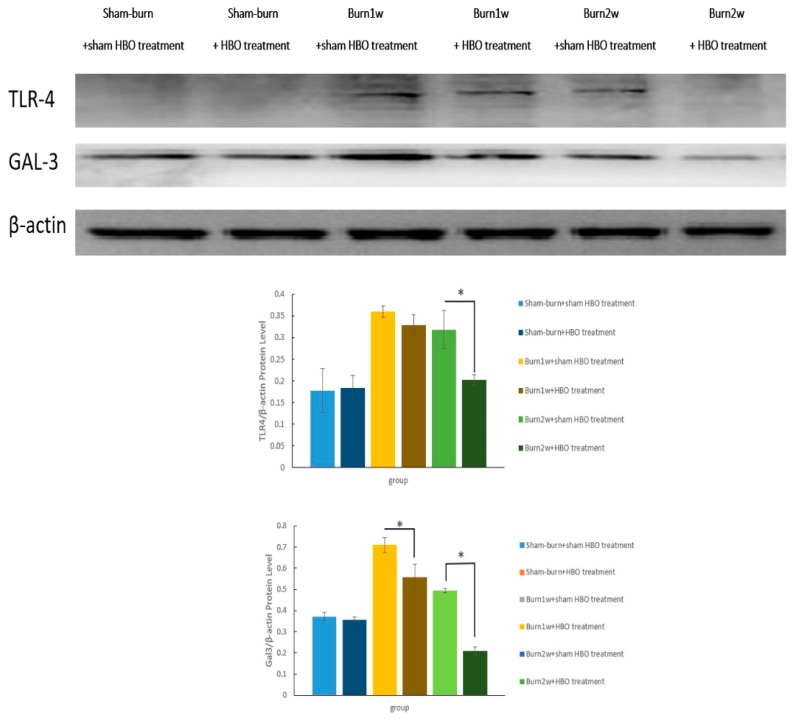
HBO treatment inhibited the expression of the inflammatory proteins GAL-3 and TLR-4 in the spinal cord dorsal horn. Western blot analyses of TLR-4 and GAL-3 in the spinal cord dorsal horn at one and two weeks after burn injury. β-actin was used as an internal control. Protein expression of GAL-3 decreased significantly in the burn with HBO treatment group compared with the burn with sham HBO treatment group (* *p* < 0.05), and protein expression of TLR-4 decreased significantly in the burn with two-week HBO treatment group compared with the burn with two-week sham HBO treatment group (* *p* < 0.05).

**Figure 6:**
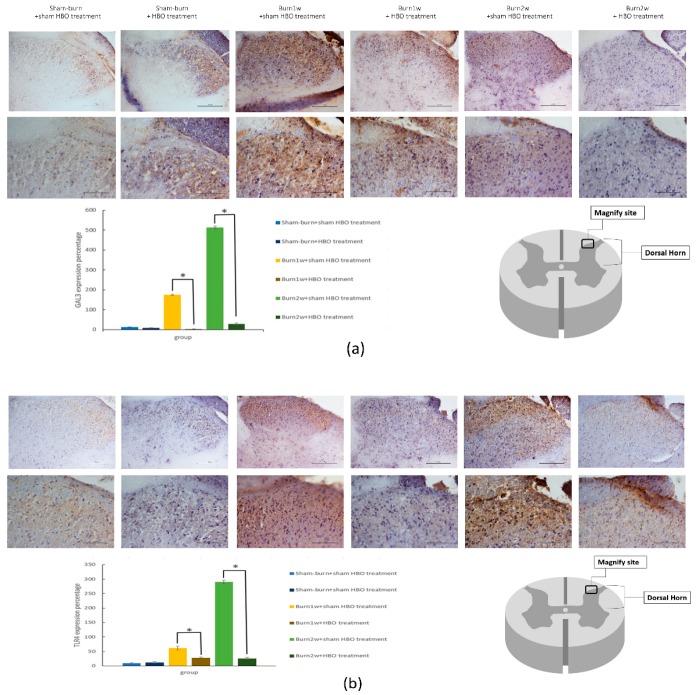
(**a**,**b**) HBO treatment inhibited the expression of GAL-3 and TLR-4 in spinal cord right dorsal horn histologic specimens. Immunohistochemistry (IHC) analyses of GAL-3 and TLR-4 in the spinal cord dorsal horn at one and two weeks after burn injury. GAL-3 and TLR-4 expression decreased significantly in the burn with HBO treatment group compared with the burn with sham HBO treatment group (* *p* < 0.05), original magnification: ×20 for upper part in (**a**,**b**), original magnification: ×40 for lower part in (**a**,**b**).

To investigate the effect of HBO treatment on the Gal-3-dependent TLR-4 pathway, the expression of Gal-3 and TLR-4 in the dorsal horns of the spinal cord was assessed using Western blotting and IHC analysis. In Western blotting, β-actin was used as an internal control. The expression of Gal-3 and TLR-4 significantly decreased in the burn with two-week HBO treatment group (Figure 5). The results of the IHC analysis of Gal-3 and TLR-4 showed Gal-3 and TLR-4 expression in the right dorsal horn. HBO treatment reduced Gal-3 and TLR-4 expression in the right dorsal horn (Figure 6a,b).

### 2.4. HBO Treatment Increases Growth Factor Expression in the Hind Paw Skin of Rats with Full-Thickness Burn Injury

**Figure 7:**
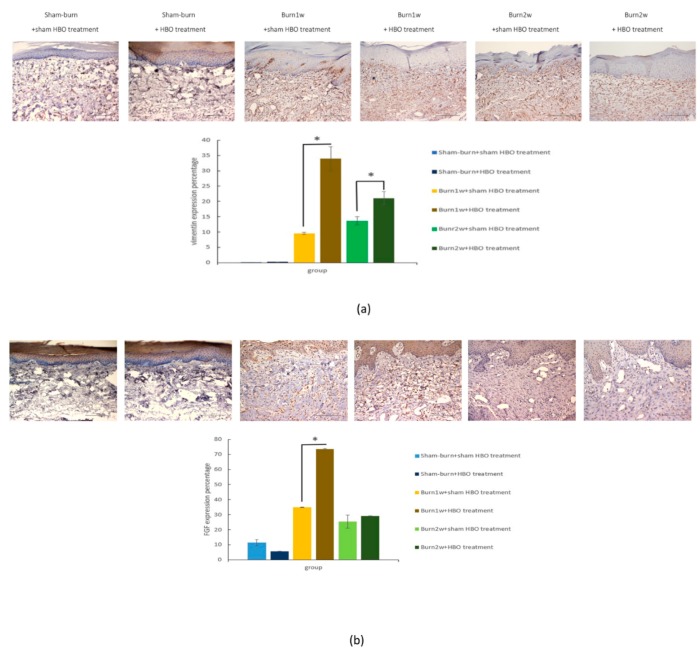
(**a**,**b**) HBO treatment increased vimentin and fibroblast growth factor expression. Immunohistochemistry (IHC) analyses of vimentin and fibroblast growth factor (FGF) in hind paw skin histologic specimens at one and two weeks after burn injury. Vimentin expression increased significantly in the burn with HBO treatment group compared with the burn with sham HBO treatment group. FGF expression increased in the burn with one-week HBO treatment group compared with the burn with one-week sham HBO treatment group. * *p* < 0.05, original magnification: ×20.

To confirm whether HBO treatment promotes regeneration of the full-thickness burn injury wound, the expression of vimentin and fibroblast growth factor (FGF) in the right hind paw skin was assessed using IHC analysis. Vimentin expression significantly increased in the burn with HBO treatment group, and FGF expression significantly increased in the burn with one-week HBO treatment group (Figure 7a,b). According to the IHC analysis results, HBO treatment can promote full-thickness burn injury wound regeneration.

## 3. Discussion

**Figure 8:**
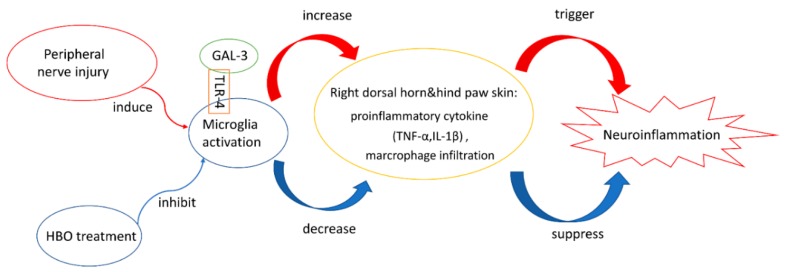
HBO treatment suppressed neuroinflammation by inhibiting the Gal-3-dependent TLR-4 pathway. HBO treatment inhibited GAL-3 and TLR-4 expression. Moreover, HBO treatment decreased proinflammatory cytokine expression and macrophage infiltration in the right dorsal horn and the right hind paw skin.

Our study results revealed that HBO treatment in the early stages after burns effectively attenuated burn-induced neuroinflammation in rats by inhibiting the Gal-3-dependent TLR-4 pathway (Figure 8). HBO therapy is widely used as an effective and noninvasive method for various diseases. HBO treatment reduces healing time and improves outcomes. In 1965, HBO treatment was used to heal second-degree burns in coal miners [41]. Based on the results of previous studies and the findings of the present study, HBO treatment may be a novel nonpharmacological approach for reducing burn-induced neuroinflammation. Nevertheless, the efficacy of long-term HBO treatment has yet to be investigated.

Burn injury breaks the intactness of the local skin, which increases the risk of infection. Moreover, burn injury can induce neuroinflammation and immunosuppression [42,43,44]. In the peripheral nervous system (PNS) and central nervous system (CNS), neuroinflammation triggers the activation of glial cells, which include satellite glial cells, astrocytes, and microglial cells [45]. Neuroinflammation also causes immune cells, such as macrophages and neutrophils, in the circulation to be recruited to the PNS and CNS [46].

Microglial cells originate from myeloid precursors in the yolk sac, and they are macrophages of the CNS [47]. Microglia can be induced by LPS or injury, and migrate from blood to the brain through the blood–brain barrier (BBB) [48]. Microglia play an important role as responders to injury or pathogens, and modulate inflammatory cytokine production. Proinflammatory cytokines, including TNF-α and IL-1β [49], activate endothelial cells [50], which in turn increase the leukocyte-endothelial interactions and assist in the recruitment of immune cells across the blood-brain barrier (BBB) to the brain [51].

Our previous study indicated that full-thickness burn injury can induce microglial cell activation in an animal model. After nerve injury or burns, the activation of microglia plays an important role in pain development and maintenance [52]. Our previous study also showed a higher expression of p38 in a burn injury rat model [40]. Gal-3 modulates neutrophil activation through p38 phosphorylation [53]. Gal-3 is distributed in many tissues, such as the heart, lungs, blood, kidneys, and digestive tract. Monocytes, macrophages, dendritic cells, and epithelial and endothelial cells exhibit Gal-3 expression [54,55,56,57]. After stimulation with pathogens or LPS, Gal-3 can be detected both intracellularly and extracellularly [58,59]. Gal-3 is involved in both inflammation and neuropathic pain [27,28,29,30]. TLR-4 is correlated with noninfectious inflammatory diseases. TLR-4 expression increases with an increase in cytokine production [60,61]. The Gal-3-dependent TLR-4 pathway is related to the inflammatory response in the brain [30,62]. Thus, we assume that burn-induced neuroinflammation is also related to the Gal-3-dependent TLR-4 pathway.

Our results indicated that the MWT significantly increased after HBO treatment. Both the burn with one-week HBO treatment and burn with two-week HBO treatment groups showed significantly higher MWT values than the burn without HBO treatment group. However, no difference was observed in TWL between the burn with HBO treatment group and the burn with sham HBO treatment group.

The immunoblotting and IHC analysis results showed that HBO treatment in the early stages of full-thickness burn injury reduced the expression of CD45, CD68, Gal-3, and TLR-4 in the right dorsal horn. ELISA results also showed significantly decreased levels of the proinflammatory cytokines TNF-α and IL-1β in the dorsal horn of the spinal cord and the hind paw skin.

A study showed that the activation of the Gal-3-dependent TLR-4 pathway may contribute to sustained microglial cell activation, prolonging the inflammatory response in a murine neuroinflammatory model (LPS injection) and in patients with stroke [17]. Gal-3 can act as an endogenous ligand for TLR-4 and induce the TLR-4-dependent inflammatory response; its expression is increased in microglial cells activated through various neuroinflammatory stimuli [62]. L5 spinal nerve ligation in rats leads to an increase in Gal-3 expression in the dorsal root ganglion. Gal-3 depletion exerts neuroprotective and anti-inflammatory effects following global brain ischemia and in the neuroinflammatory LPS model [17]. In accordance with these observations, increased expression of Gal-3 and TLR-4 was detected in the spinal cord of rats with burn injury. Importantly, HBO treatment reduced the expression of Gal-3 and TLR-4 in rats with burn injury, suggesting that the anti-inflammatory effects of HBO treatment may result from the suppression of the Gal-3-dependent TLR-4 pathway, which subsequently inhibits microglial cell activation and inflammation.

Activation of microglia and macrophages results in the development of two phenotypes: proinflammatory (M1) and anti-inflammatory (M2) [63]. M1 microglia synthesize and secrete large amounts of reactive oxygen species (ROS) and proinflammatory cytokines, including IL-1β, IL-6, and TNF-α [64,65]. TNF-α and IL-1β production is mainly induced by activated macrophages [66]. CD45 and CD68 are microglia and macrophage markers. The IHC analysis revealed that HBO treatment inhibited microglia and macrophage expression and recruitment in the right dorsal horn. Consequently, HBO treatment reduced proinflammatory cytokine production by inhibiting microglia and macrophage activation and accumulation in the right dorsal horn.

Macrophage MIF is a chemokine-like proinflammatory cytokine that promotes leukocyte recruitment and the secretion of nitric oxide or inflammatory cytokines, such as TNF-α, IL-1β, and IFN-γ [67,68]. After stimulation with LPS or inflammatory cytokines, CD68 expression in macrophages significantly increased [69,70]. Thus, MIF and CD68 can be used as markers to detect both M1 and M2 macrophages and reveal macrophage expression of the burn-injury site [71,72]. After HBO treatment, the expression of TNF-α and IL-1β significantly decreased. Moreover, the IHC analysis revealed lower CD68 and MIF expression in our HBO treatment groups. This finding illustrates that HBO treatment suppresses macrophage recruitment and the infiltration of the wound site with various inflammatory cells.

Research has indicated that vascular endothelial growth factor, platelet-derived growth factor, and FGF were upregulated after HBO treatment [73]. A previous study demonstrated that HBO treatment enhanced the proliferation of modified Brain-derived neurotrophic factor (NIH3T3/BDNF) fibroblasts [74]. In an ischemic mice model, HBO treatment increased the production of basic FGF and accelerated muscle regeneration [75]. In the present study, after HBO treatment, the expression of vimentin and FGF significantly increased in the right hind paw skin. Therefore, HBO treatment improved wound regeneration in a burn injury rat model.

In clinical settings, HBO treatment for burn injury should be initiated in the first 24 h. The pressure within the chamber should be 2.0–2.4 ATA for 90 min, HBO treatment should be administered twice daily, and the number of treatments should depend on the response to treatment [41].

Our results are consistent with the aforementioned findings; yet, some issues remain. Whether Gal-3 or TLR-4 overexpression can reverse the effects of HBO treatment remains to be investigated. Moreover, the length of time for which the effects of HBO treatment can persist should be assessed. Few studies have discussed this issue and have shown that, in a murine CCI injury model, a single HBO treatment caused a short-acting antinociceptive response phase (less than two hours). Repetitive HBO treatment (daily for seven consecutive days) led to a longer effect (more than 24 h) [23]. Additional studies should use Gal-3 or TLR-4 inhibitors combined with HBO treatment and should evaluate the length of time for which the effects of HBO treatment on burn injury are sustained.

## 4. Materials and Methods

### 4.1. Animal Preparation and Experimental Design

Thirty-six adult male Sprague-Dawley (SD) rats, weighing 160–180 g, were used in this study. All rats were housed in a humidity- and temperature-controlled room (55% ± 15% and 22 °C ± 1 °C, respectively) under a 12-h light-dark cycle. All experimental animals were provided standard amounts of food and water. This study was approved by the Institutional Animal Care and Use Committee of Kaohsiung Medical University (IACUC 01/07/2017 no. 106078). Rats were divided into the following six groups (*n* = 6 per group): (1) sham burn with sham HBO treatment; (2) sham burn with HBO treatment: normal rats were sacrificed after 1-week HBO treatment; (3) burn with 1-week sham HBO treatment: experimental animals were sacrificed at 1 week after burn injury; (4) burn with 2-week sham HBO treatment: experimental animals were sacrificed at 2 weeks after burn injury; (5) burn with 1-week HBO treatment: after burn injury, experimental animals were sacrificed 1 week after HBO treatment (which was provided daily); and (6) burn with 2-week HBO treatment: after burn injury, experimental animals were sacrificed after 2-week HBO treatment (which was provided daily).

### 4.2. Full-Thickness Burn Injury Model

To establish a third-degree burn injury model, rats were anesthetized through a subcutaneous injection with Zoletil 50 (50 μg/g; Virbac Laboratories, Carros, France). A metal block heated to approximately 75 °C ± 0.5 °C was then placed on the right hind paw. A 100-g weight was placed on top of the paw to maintain constant contact between the plantar surface and the metal block for 10 s. After inducing the burn injury, rats received drugs for pain control, and silver sulfadiazine cream was applied to their paws until their wounds healed.

### 4.3. HBO Treatment

An HBO treatment chamber (Genmall Biotechnology Co., Ltd., Taipei, Taiwan) was used. After experimental rats were placed in the chamber, the pressure was increased to the desired pressure (2.5 ATA) in 20 min, and that pressure was maintained for 1 h. Rats were allowed to breathe freely during HBO treatment. The chamber was then decompressed to normal room pressure in 20 min. On the next day after burn injury, rats in the HBO treatment groups received HBO treatment once a day.

### 4.4. Behavior Test

Rat behaviors were observed 1 day before burn injury and 7 and 14 days after burn injury. Mechanical thresholds were measured based on the withdrawal of the paw with burn injury and were tested using a Dynamic Plantar Aesthesiometer (Ugo Basile, Comerio, Italy). The mid-plantar surface of the paw was placed on a metal mesh, and pressure at a rate of 2.5 g/s was applied to a metal rod (2-mm diameter) until the animal withdrew the paw. The response to noxious heat was measured using a Hargreaves apparatus (Model 7370; Ugo Basile), which projected a beam of infrared light to heat a glass plate upon which the paw was placed. The time from application of the heat source to withdrawal of the hind paw was measured. Each measurement was repeated five times at 10-min intervals, and rats were allowed 30 min of rest between hind paw applications.

### 4.5. Western Blot Assay

Tissue lysates were homogenized in Complete Protease Inhibitor Cocktail (Roche Molecular Systems, Inc., Pleasanton, CA, USA) using a Magna Lyser (Roche Molecular Systems). Proteins at 40 μg/well were loaded and run on 12% sodium dodecyl sulfate polyacrylamide gel electrophoresis (SDS PAGE); the separated proteins were blotted onto a polyvinylidene difluoride (PVDF) membrane. The membrane was then blocked with 5% defatted milk powder. After blocking, the membrane was incubated with polyclonal rabbit antirat TLR-4 and GAL-3 antibodies (1:500; Novus Biologicals, LLC, Littleton, CO, USA) and polyclonal mouse antirat β-actin antibodies (1:1000; Cell Signaling Technology, Inc., Beverly, MA, USA), followed by incubation with suitable horseradish peroxidase (HRP)-conjugated secondary antibodies. Immunoreactive bands were visualized using the ChemiDoc XRS+ System (Bio-Rad Laboratories, Inc., Hercules, CA, USA).

### 4.6. Immunohistochemical Assay

After antigen retrieval at a high pH for 20 min, paraffin-embedded sections were incubated with anti-TLR-4 (1:100; Novus Biologicals, LLC), anti-GAL-3 (1:100; Novus Biologicals, LLC), anti-MIF (1:200; Abcam, Cambridge, UK), anti-CD45 (1:100; Abcam, Cambridge, UK), anti-CD68 (1:100; Abcam, Cambridge, UK), anti-vimentin (1:200; Cell Signaling Technology, Inc.), and anti-fibroblast growth factor-basic (1:500; Sigma-Aldrich, Merck KGaA, Darmstadt, Germany). The sections were then washed with Tris-buffered saline and incubated with the biotinylated secondary antibody Histofine Simple Stain Rat MAX PO (Nichirei Bioscience, Nichirei Corporation, Tokyo, Japan.), and the diaminobenzidine substrate was added to react with horseradish peroxidase (HRP) following the protocol of Dako REAL Detection System, Peroxidase/DAB+, Rabbit/Mouse (Dako Products, Santa Clara, CA, USA). Finally, the sections were stained with hematoxylin. Semiquantitative analysis of immunostained slides was performed using Image-Pro Plus Version 6.0 (Media Cybernetics, Inc., Rockville, MD, USA).

### 4.7. Enzyme-Linked Immunosorbent Assay

TNF-α and IL-1β concentrations in experimental animal tissue lysates were measured using rat TNF-α and IL-1β enzyme-linked immunosorbent assay (ELISA) kits (eBioscience, Thermo Fisher Scientific Inc., Waltham, MA, USA. An ELISA plate was coated with 100 μL of a capture antibody in a coating buffer and was incubated overnight at 4 °C. The plate was then washed with a wash buffer three times and blocked with 300 μL of a blocking buffer for 1 h at room temperature. After blocking, the plate was washed three times with a wash buffer. Standards were diluted to concentrations of 2000, 1000, 500, 250, 125, 62.5, 31.25 and 0 pg/mL. The standards or samples (100 μL) were added to each well in triplicate and were incubated for 2 h at room temperature. Subsequently, the ELISA plate was washed three times, incubated with 100 μL of a detection antibody for 1 h at room temperature, and washed three times. The plate was incubated with avidin- horseradish peroxidase (HRP) conjugates (1:1000; 100 μL) for 30 min and then washed five times. Finally, freshly mixed Tetramethylbenzidine (TMB) substrate solution (100 μL) was added to each well and incubated in the dark for 30 min. Stop solution (2N H_2_SO_4_; 100 μL) was added to each well, and within 15 min of stop solution addition, absorbance at 450 nm was read on a Multiskan Ascent 96/384 Plate Reader (MTX Lab Systems, Bradenton, FL, USA).

### 4.8. Statistical Analysis

The numerical data are expressed as means with standard deviation (SD). Statistical analysis was performed using one-way ANOVA on SPSS 14.0 software (SPSS, Inc., Chicago, IL, USA). *p* < 0.05 was considered statistically significant.

## 5. Conclusions

Our data indicated that early HBO treatment inhibits the Gal-3-dependent TLR-4 pathway. This study also proved that HBO treatment suppresses microglia/macrophage activation following burn injury and promotes wound regeneration. The present study revealed that HBO treatment is effective for burn injury and neuroinflammation. According to the behavior test result, HBO treatment significantly increases the MWT. This finding illustrates that HBO treatment may be useful for ameliorating burn injury-induced neuropathic pain.
